# Proteomic Profiling of the Interactions of Cd/Zn in the Roots of Dwarf Polish Wheat (*Triticum polonicum* L.)

**DOI:** 10.3389/fpls.2016.01378

**Published:** 2016-09-14

**Authors:** Yi Wang, Xiaolu Wang, Chao Wang, Ruijiao Wang, Fan Peng, Xue Xiao, Jian Zeng, Xing Fan, Houyang Kang, Lina Sha, Haiqin Zhang, Yonghong Zhou

**Affiliations:** ^1^Triticeae Research Institute, Sichuan Agricultural UniversitySichuan, China; ^2^College of Resources, Sichuan Agricultural UniversitySichuan, China

**Keywords:** dwarf polish wheat, iTRAQ, cadmium, zinc, interaction, proteomic

## Abstract

Cd and Zn have been shown to interact antagonistically or synergistically in various plants. In the present study of dwarf polish wheat (DPW)roots, Cd uptake was inhibited by Zn, and Zn uptake was inhibited by Cd, suggesting that Cd and Zn interact antagonistically in this plant. A study of proteomic changes showed that Cd, Zn, and Cd+Zn stresses altered the expression of 206, 303, and 190 proteins respectively. Among these, 53 proteins were altered significantly in response to all these stresses (Cd, Zn, and Cd+Zn), whereas 58, 131, and 47 proteins were altered in response to individual stresses (Cd, Zn, and Cd+Zn, respectively). Sixty-one differentially expressed proteins (DEPs) were induced in response to both Cd and Zn stresses; 33 proteins were induced in response to both Cd and Cd+Zn stresses; and 57 proteins were induced in response to both Zn and Cd+Zn stresses. These results indicate that Cd and Zn induce differential molecular responses, which result in differing interactions of Cd/Zn. A number of proteins that mainly participate in oxidation-reduction and GSH, SAM, and sucrose metabolisms were induced in response to Cd stress, but not Cd+Zn stress. This result indicates that these proteins participate in Zn inhibition of Cd uptake and ultimately cause Zn detoxification of Cd. Meanwhile, a number of proteins that mainly participate in sucrose and organic acid metabolisms and oxidation-reduction were induced in response to Zn stress but not Cd+Zn stress. This result indicates that these proteins participate in Cd inhibition of Zn uptake and ultimately cause the Cd detoxification of Zn. Other proteins induced in response to Cd, Zn, or Cd+Zn stress, participate in ribosome biogenesis, DNA metabolism, and protein folding/modification and may also participate in the differential defense mechanisms.

## Introduction

Environmental toxicity from non-essential heavy metals such as cadmium (Cd), which is released from human activities and other environmental causes, is rapidly increasing (Ahsan et al., 2009). In humans, Cd causes diseases such as osteoporosis and emphysema by damaging the lungs, kidneys, and bones (Kazantzis, 2004; Straif et al., 2009). In plants, Cd damages the photosynthetic apparatus, interrupts respiratory and nitrogen metabolism, and unbalances water and nutrient uptake (Herbette et al., 2006; Balen et al., 2011), ultimately reducing biomass, causing leaf chlorosis, inhibiting root growth, and even leading to plant death (Lin et al., 2007; Yadav, 2010; Lin and Arats, 2012). Additionally, plants can accumulate high Cd contents in their edible parts, which poses a potentially major hazard to human health (Satarug et al., 2003).

Zinc (Zn), an essential metal and a cofactor of numerous plant proteins and enzymes, plays several crucial roles in protein binding, enzyme activity, transcriptional and translational regulation, and signal transduction (Broadley et al., 2007; Lin and Arats, 2012). However, excess Zn can also cause toxicity, as it can damage DNA replication and disrupt enzyme activities and protein folding and function, ultimately inducing chlorosis and inhibiting plant growth and development (Broadley et al., 2007; Lin and Arats, 2012; Schneider et al., 2013).

Cd and Zn coexist naturally in the soil. Due to their physical and chemical similarities (Chesworth, 1991), their uptake and transport in plants use similar pathways (Grant et al., 1998). Many metal transporters that transport both Cd and Zn have been identified, including AtNRAMP3 and AtNRAMP4 (Thomine et al., 2000; Lanquar et al., 2010). In response to Cd and Zn stresses, plants have developed strategies to prevent Cd-induced damage and maintain Zn homeostasis. Therefore, researchers have investigated the various synergistic and/or antagonistic interactions of Cd/Zn and found these interactions to depend on species, external bioavailable metal concentration, tissue type, and developmental stage. For example, some durum and bread wheat show antagonistic interactions of Cd/Zn in which Cd uptake is inhibited by Zn and Zn uptake is inhibited by Cd in roots, stems, and leaves (Hart et al., 2002, 2005; Sun et al., 2005). Conversely, some wheat under field conditions has shown synergistic interactions in which Cd and Zn uptake are promoted by each other (Nan et al., 2002).

However, previous studies on Cd/Zn interactions mainly focused on their transport and biochemical responses (Hart et al., 2002, 2005; Nan et al., 2002; Hassan et al., 2005; Sun et al., 2005). Although proteomic changes in response to Cd or Zn have been successfully investigated using a proteomics approach (Kieffer et al., 2008, 2009; Ahsan et al., 2009; Fukao et al., 2011; Lin and Arats, 2012; Schneider et al., 2013), the molecular mechanisms of Cd/Zn interactions are unknown, which limits our understanding of the interactions of Cd/Zn. Polish wheat (2*n* = 4x = 28, AABB, *Triticum polonicum* L.), which has low genetic similarity with *T. aestivum* (Wang et al., 2013; Michalcová et al., 2014), accumulates high concentrations of Zn, Fe, and Cu and therefore has attracted the interest of producers and breeders (Wiwart et al., 2013). Meanwhile, dwarf polish wheat (DPW), collected from Tulufan, Xingjiang, China, shows high tolerance to Cd and Zn because its growth is not affected by the accumulation of high concentrations of these metals in seedlings (Wang X. et al., in press). However, molecular responses to Cd and Zn remain unknown. Since DPW accumulates high concentrations of Cd and Zn in seedlings, it is a useful system for studying Cd/Zn interactions.

The purposes of this study are to understand molecular responses to Cd and Zn stresses, to investigate Cd/Zn interactions in DPW seedlings and to understand the molecular mechanisms of Cd/Zn interactions in DPW roots using isobaric tags for relative and absolute quantification (iTRAQ). iTRAQ is a high-throughput proteomic technology (Karp et al., 2010) that has been successfully used to reveal plant responses to heavy metals (Ahsan et al., 2009; Fukao et al., 2011).

## Materials and methods

### Plant material and growth conditions

DPW seeds were sterilized with 1% NaOCl and germinated in the dark for 5 days. The seedlings were cultured in full Hoagland nutrient solution in a growth chamber at 25°C with a 16 h-light/8 h-dark cycle. At the two leaf stage, the seedlings were treated with null (CK), 40 μM CdSO_4_ (Cd), 800 μM ZnCl_2_ (Zn), or 40 μM CdSO_4_ + 800 μM ZnCl_2_ (Cd+Zn). Two days after treatments, the roots (three biological replications, each replication including 15 plants) were washed with 0.1 μM EDTA and ddH_2_O, snap frozen in liquid nitrogen and stored at −80°C for iTRAQ analysis. Other roots and leaves were dried for 2 days at 80°C for measuring metal concentrations.

### Measurement of Cd and Zn concentrations

Cd and Zn concentrations were measured as described by Wang et al. (2014). Briefly, the dried roots and leaves were ground to particle powders. Then, 0.2 g of powder was digested using concentrated sulfuric acid and hydrogen peroxide at 320°C and then diluted to 50 ml. Metal concentrations were then determined using an atomic absorption spectrometer, Analyst 400 (PerkinElmer, CT, USA). Standard solutions of Cd and Zn were purchased from Fisher Scientific Ltd. (China). All data and figures were analyzed (*t*-test was conducted for the statistical analysis) and drawn using Sigmaplot 12.0.

### Total protein extraction

Roots (two randomly selected biological replications) with 0.1 mg of polyvinylpyrrolidone (PVPP) were ground into powders using liquid nitrogen and then homogenized in Tris-phenol (pH 8.0) and protein extraction buffer (0.7 M sucrose, 0.1 M KCl, 50 mM EDTA, 0.5 M Tris, pH 7.5, 2% β-mercaptoethanol, and 1 mM PMSF). After centrifuging for 20 min at 6000 rpm, the supernatants were collected and re-purified using protein extraction buffer. Proteins were precipitated using ammonium acetate methanol and then washed with methanol and acetone. Finally, protein samples were diluted using RIPA reagent, and protein concentrations were measured using a BCA Assay Kit (Biotech).

### iTRAQ labeling and LC-MS analysis

iTRAQ labeling was performed according to Wu et al. (2013) with modifications. Briefly, 200 μg of protein from each sample (two biological replications) was reduced, alkylated and then subjected to tryptic hydrolysis. iTRAQ labeling was performed using an iTRAQ® reagents-8plex Kit (Applied Biosystems). Peptides of CK, Cd, Zn, and Cd+Zn samples were labeled singly with the iTRAQ reporters 113, 114, 115, and 116, respectively. LC-MS (TripleTOF5600, Applied Biosystems) analysis was performed as described by Wu et al. (2013).

### Protein identification and quantification

Protein identification and relative quantification were also performed according to Wu et al. (2013). Protein Pilot software v. 4.0 (Applied Biosystems) was used to convert the raw data (.wiff) into peak lists (.mgf). Each MS/MS spectrum was searched against the protein database Uniprot-147389. The search parameters were as follows: Paragon method: iTRAQ-8plex, Cys alkylation: MMTS, Digestion: Trypsin, Instrument: TripleTOF 5600, ID focus: Biological modifications and Amino acids substitutions, Detected Protein Threshold [Unused ProtScore (Confidence)]: ≥ 1.3, Competitor Error Margin (ProtScore): 2.0, and No. Distinct Peptides (Confidence): ≥ 95%. The tolerances were specified as ± 0.05 Da for peptides and ± 0.05 Da for MS/MS fragments. The relative abundance (fold-change ratios of differential abundance between labeled samples), *P*-value, error factor, lower confidence interval and upper confidence interval were calculated using the ProteinPilot software. Proteins containing at least two distinct peptides and fold change ratios ≥ 1.5 or ≤ 0.67 were considered as more abundant or less abundant proteins, respectively.

## Results

### Metal concentrations

**Figure 1:**
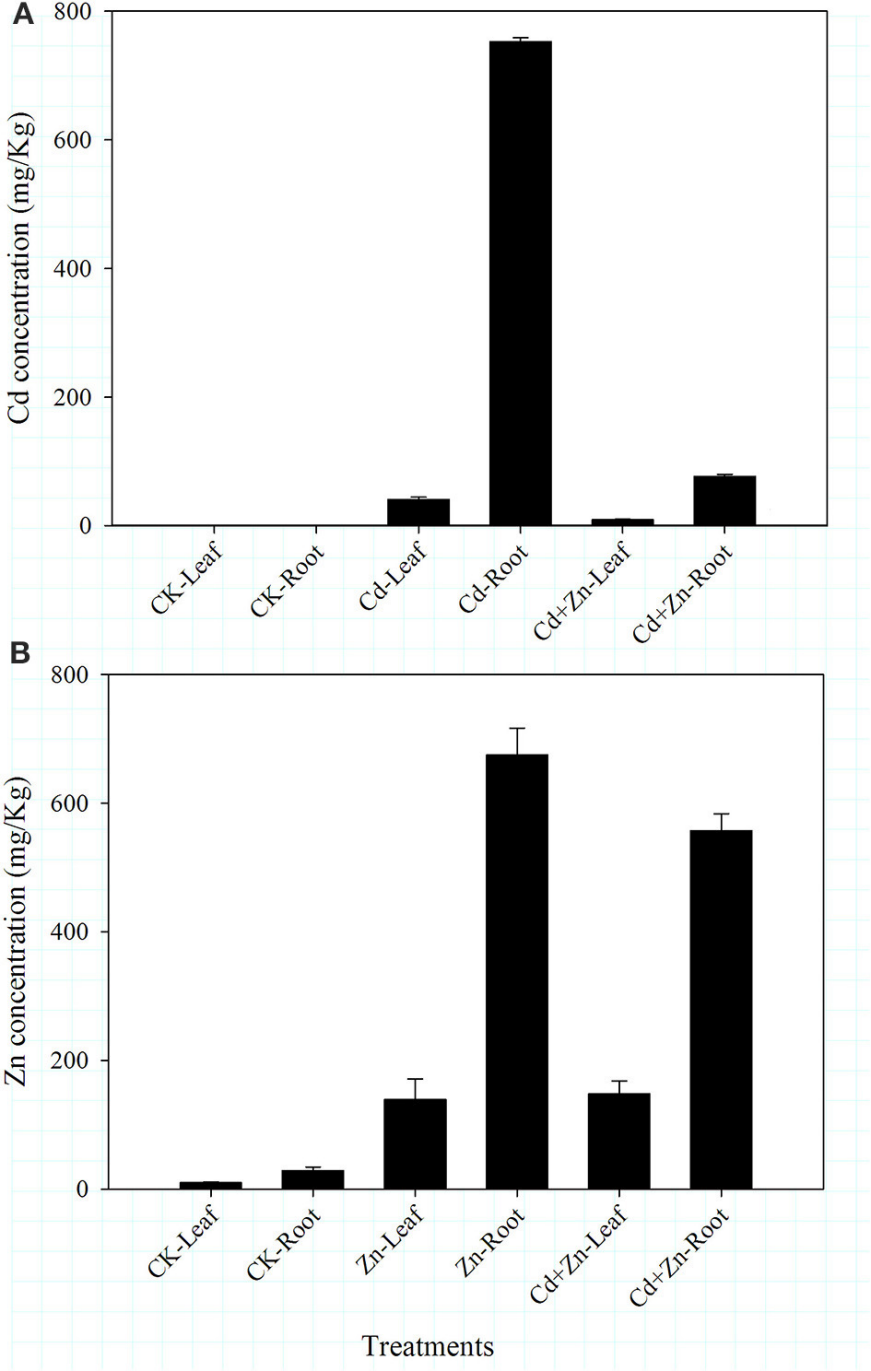
**Metal concentrations in roots and leaves 2 days after treatment. (A)** Cd concentrations; **(B)** Zn concentrations. Values were means ± standard error (three biological replications).

No Cd was detected in CK (control) samples (Figure 1A). Two days after treatment, the Cd concentration in roots treated with Cd (752.55 ± 6.51 mg/Kg) was significantly higher (*P* < 0.01) than that in roots treated with Cd+Zn (76.75 ± 3.312 mg/Kg; Figure 1A). Meanwhile, the Cd concentration in leaves under Cd stress (40.87 ± 3.69 mg/Kg) was also significantly higher (*P* < 0.01) than that in leaves under Cd+Zn stress (9.20 ± 1.24 mg/Kg; Figure 1A). These results indicate that Zn inhibits Cd uptake in roots as well as its transport from roots to shoots.

Zn concentrations in the roots were always higher than those in the leaves (Figure 1B). Zn concentrations in leaves were similar between Zn (139.26 ± 32.12 mg/Kg) and Cd+Zn (147.00 ± 20.15 mg/Kg) stresses (Figure 1B). In roots treated with Zn (675.36 ± 41.67 mg/Kg), the Zn concentration was significantly higher (*P* < 0.01) than that in roots treated with Cd+Zn (557.63 ± 26.30 mg/Kg; Figure 1B). These results suggest that Cd inhibits Zn uptake in roots but does not affect its transport from root to shoot.

### A total of 432 proteins were altered by Cd, Zn, or Cd+Zn stresses

**Figure 2:**
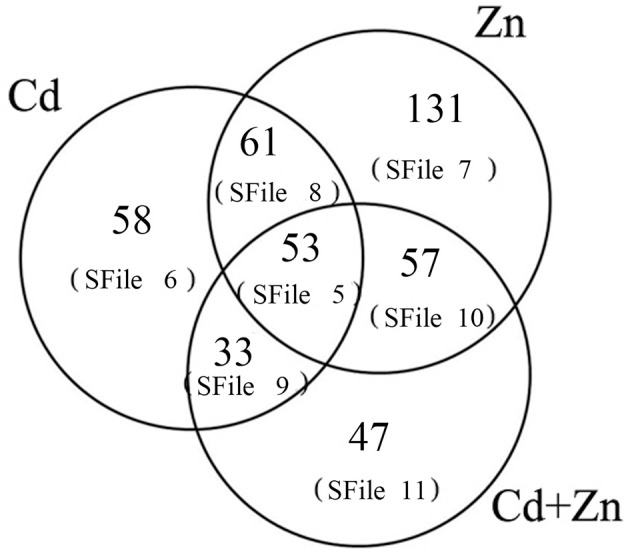
**Numbers of altered proteins which were classified into differentially interactive groups in response to Cd, Zn, and Cd+Zn stresses**.

A total of 960 proteins with one or more distinct peptides and an Unused ProtScore ≥ 1.3 (with a peptide confidence ≥ 95%) (Data Sheet 1) were identified from the protein database Uniprot-147389. Compared with null, the expression levels of 206 (Data Sheet 2), 303 (Data Sheet 3), and 190 (Data Sheet 4) proteins were altered by Cd, Zn, and Cd+Zn stresses, respectively. Further analysis indicated that these proteins could be grouped into seven sub-groups (Table 1, Figure 2).

**Table 1:** **Some noteworthy proteins identified in differentially interactive groups**.

**ID^a^**	**Name**	**UP^b^**	**No. P^c^**	**Ratio**		
				**CK/Cd**	**CK/Zn**	**CK/Cd+Zn**
**PROTEINS WERE INDUCED IN ALL OF Cd, Zn, And Cd+Zn STRESSES**						
**Sucrose metabolism**						
466	Beta-1,3-glucanase	4.00	2	0.49	0.38	0.24
4	Beta-glucosidase	26.16	26	2.09	2.68	2.73
675	Beta-glucosidase	2.42	21	4.79	22.91	2.75
779	Glucan endo-1,3-beta-glucosidase 12	2.07	2	0.40	0.39	0.44
554	Glucose-6-phosphate isomerase	3.54	2	0.50	1.61	2.63
**GSH metabolism**						
540	Sulfurtransferase	3.61	2	9.12	1.74	3.13
112	ATP sulfurylase	8.47	5	0.18	0.32	0.53
424	Lactoylglutathione lyase	4.05	2	1.53	0.39	0.61
405	Glutathione-S-transferase	4.06	4	0.42	0.22	0.38
141	Glutaredoxin	8.00	5	0.16	0.12	0.12
**Oxidation-reduction process**						
908	Cationic peroxidase SPC4	2.00	14	0.53	0.61	0.63
248	Peroxidase 66	6.01	33	0.62	0.41	0.54
616	Cytochrome c oxidase subunit 6B	2.98	2	0.50	8.55	0.65
**PROTEINS WERE INDUCED ONLY IN Cd And Zn STRESSES**						
**Oxidation-reduction process**						
230	NADH dehydrogenase	4	4	1.96	3.02	0.70
144	Peroxidase 12	6.12	19	0.57	0.54	1.18
**Sucrose metabolism**						
872	Beta-fructofuranosidase	2.01	3	0.62	0.39	1.05
**SAM metabolism**						
258	Serine hydroxymethyltransferase	6.00	3	0.43	0.27	1.34
677	Spermidine synthase 1	2.41	2	4.70	1.72	1.07
**PROTEINS WERE INDUCED ONLY IN Cd And Cd+Zn STRESSES**						
**SAM metabolism**						
519	Nicotianamine synthase 2	3.89	2	0.56	1.05	1.60
748	S-adenosylmethionine synthase 1	2.11	13	2.31	1.14	1.54
**Ca metabolism**						
306	Calmodulin-related protein	5.07	3	0.65	1.29	0.46
**Sucrose metabolism**						
371	Xylose isomerase	4.19	3	3.05	0.67	3.70
**PROTEINS WERE INDUCED ONLY IN Cd STRESS**						
**Oxidation-reduction process**						
285	Ascorbate peroxidase	5.52	19	0.66	1.33	0.67
552	NADH dehydrogenase iron-sulfur protein 4	3.55	2	2.27	1.26	0.77
313	Peroxidase 1	4.92	3	0.30	0.83	0.69
356	Peroxidase 12	4.32	2	2.25	1.03	1.43
5	Peroxidase 12	25.81	28	0.64	0.89	0.80
775	Peroxidase 2	2.08	2	0.62	1.12	0.69
13	Root peroxidase	20.37	27	8.71	0.86	1.10
1185	Frataxin, mitochondrial	2.00	2	28.58	0.81	0.85
**GSH metabolism**						
210	Glutamine synthetase cytosolic isozyme 1-2	6.30	3	0.62	1.24	0.74
696	Glutathione S-transferase GSTU6	2.26	5	0.65	0.92	0.90
135	Lactoylglutathione lyase	8.00	6	1.71	1.06	1.06
**Sucrose metabolism**						
1111	Polygalacturonase	2.00	2	0.59	0.71	0.93
357	Beta-amylase	4.31	3	0.58	0.80	0.82
**Ca metabolism**						
44	Calreticulin-like protein	12.57	9	0.59	1.26	0.82
430	Calcium-binding protein CML27	4.04	2	0.54	1.12	0.70
316	Calcium-dependent protein kinase	4.86	2	0.60	0.77	1.49
**SAM metabolism**						
410	Caffeic acid 3-O-methyltransferase	4.06	2	0.64	1.02	0.95
215	Serine hydroxymethyltransferase 1	6.26	3	2.25	1.41	1.39
**PROTEINS WERE INDUCED ONLY IN Zn And Cd+Zn STRESSES**						
**GSH metabolism**						
731	Glutathione S-transferase GSTU6	2.14	2	0.77	0.39	0.56
79	Glutathione-S-transferase 28e45	10.21	13	1.16	0.32	0.33
384	Glutaredoxin-S4, mitochondrial	4.12	3	1.14	0.28	0.49
472	Glutaredoxin-C8	4.00	2	1.08	1.60	0.63
**Oxidation-reduction process**						
440	Peroxidase 12	4.01	24	0.97	0.39	0.63
102	Peroxidase 2	9.16	7	1.17	1.92	1.77
890	Peroxidase 52	2.00	17	0.76	0.38	0.63
**Sucrose metabolism**						
603	Glucan 1,3-beta-glucosidase	3.09	2	0.81	0.17	1.98
**PROTEINS WERE INDUCED ONLY IN Zn STRESS**						
**Sucrose metabolism**						
298	6-phosphogluconate dehydrogenase	5.24	4	1.22	1.54	0.90
21	Alpha-1,4-glucan-protein synthase	17.82	10	1.20	1.51	0.87
366	Alpha-L-arabinofuranosidase 1	4.21	3	1.16	2.38	1.08
1056	Beta-glucanase	2.00	3	0.92	0.60	1.42
741	Fructose-bisphosphate aldolase 3	2.13	3	0.86	0.66	1.25
8	Sucrose synthase 1	22.86	16	1.09	2.05	1.10
205	UDP-glucose 6-dehydrogenase	6.34	4	0.88	2.38	0.76
192	UTP-glucose1-phosphate uridylyltransferase	6.56	6	1.06	2.83	1.04
**Organic acids metabolism**						
178	2-oxoglutarate dehydrogenase	6.88	4	0.86	1.63	1.15
58	Malate dehydrogenase	11.27	12	0.91	1.85	1.39
225	Isocitrate dehydrogenase [NADP]	6.15	4	1.43	1.56	1.26
39	Aconitate hydratase	13.06	8	1.27	1.51	1.17
82	Citrate synthase 4	10.14	7	0.77	0.59	0.73
376	Oxalate oxidase GF-2.8	4.14	2	0.91	0.35	1.21
**Oxidation-reduction process**						
573	L-ascorbate peroxidase 2, cytosolic	3.36	28	1.20	3.08	0.74
195	Lipoxygenase	6.49	5	1.25	1.74	1.46
608	Lipoxygenase	3.04	2	1.50	1.85	1.03
354	Oxidoreductase GLYR1	4.32	3	1.29	0.51	1.16
743	Peroxidase 1	2.12	6	0.80	7.52	1.21
1103	Peroxidase 12	2.00	2	0.89	0.48	1.29
651	Peroxidase 12	2.61	3	0.82	0.56	0.74
240	Peroxidase 70	6.03	8	0.79	1.61	0.68
327	Protein disulfide isomerase	4.71	4	0.70	1.61	1.07
362	Sulfite oxidase	4.26	2	0.88	0.59	0.90
689	NADH dehydrogenase (Ubiquinone) 1 alpha subcomplex subunit 5	2.28	3	0.84	2.88	0.69
**Cation transporters**						
679	P-type proton pump ATPase	2.39	2	0.95	1.66	0.77
**PROTEINS WERE INDUCED ONLY IN CD+ZN STRESS**						
**Ca metabolism**						
109	Calcium-binding protein CML7	8.62	5	1.36	1.49	0.55
**Oxidation-reduction process**						
1302	Peroxidase 2	1.40	2	1.19	1.34	1.84
245	Peroxidase 4	6.02	6	1.09	0.89	0.27
1045	Peroxidase 72	2.00	3	1.08	0.76	4.57
511	Peroxisome type ascorbate peroxidase	3.96	2	0.82	1.45	1.74
**Organic acids metabolism**						
116	Fumarate hydratase 2	8.30	4	1.22	1.16	2.29
537	Malate dehydrogenase 1	3.63	5	1.04	0.77	2.03
815	Succinate dehydrogenase	2.04	2	1.49	1.02	1.60
**Sucrose metabolism**						
50	Triosephosphate isomerase	12.00	18	0.75	0.71	1.69
786	Fructose-bisphosphate aldolase	2.06	2	1.07	0.80	2.19
187	Glucan endo-1,3-beta-glucosidase GI	6.66	6	0.97	1.00	0.61

a *represents protein identified number*,

b represents score, and

c *represents number of identified peptides*.

#### 53 Proteins were altered by all three stresses (Cd, Zn, and Cd+Zn)

The relative abundances of 53 proteins were altered significantly by all three stresses (Cd, Zn, and Cd+Zn; Figure 2, Data Sheet 5). Among these, 13 noteworthy proteins participated in either sucrose metabolism (5 proteins), glutathione (GSH) metabolism (5 proteins), or the oxidation-reduction process (3 proteins; Table 1). However, the relative abundances of other proteins were differentially altered by Cd, Zn, and combined Cd+Zn stresses (Table 1). For example, the relative abundance of glucose-6-phosphate isomerase (protein 554) was increased by Cd stress but was decreased by both Zn and Cd+Zn stresses. Contrary results were observed for lactoyglutathione lyase (protein 424), as its relative abundance was decreased by Cd stress but was increased by both Zn and Cd+Zn stresses. Further, the relative abundance of cytochrome c oxidase subunit 6B (protein 616) was increased by both Cd and Cd+Zn stresses but was decreased by Zn stress (Table 1). Thus, our analysis revealed differential molecular responses to Cd, Zn, and Cd+Zn stresses.

#### 58 Proteins were induced only in response to Cd stress

We identified 58 proteins whose relative abundances were induced only in response to Cd stress (Figure 2, Data Sheet 6). Of these, the relative abundances of 23 proteins were increased, and those of 35 proteins were decreased (Figure 2, Data Sheet 6). These proteins were not induced by either Zn or combined Cd+Zn stress, which suggests that they might participate in Zn inhibition of Cd uptake and transport. Among the 58 proteins we identified were 18 noteworthy proteins that participated in the oxidation-reduction process (4 down and 4 up), GSH metabolism (2 up and 1 down), sucrose metabolism (2 up), calcium (Ca) metabolism (3 up), or S-adenosyl-l-methionine (SAM) metabolism (1 down and 1 up; Table 1).

#### 131 Proteins were induced only in response to Zn stress

The relative abundances of 131 proteins were induced only in response to Zn stress (46 up and 85 down; Figure 2, Data Sheet 7). That these proteins were not induced in response to either Cd or combined Cd+Zn stress suggests that they might participate in Cd inhibition of Zn uptake. Among the 131 proteins we identified, we classified 26 noteworthy DEPs into four functional groups (Table 1): Sucrose metabolism (6 down and 2 up), organic acid metabolism (4 down and 2 up), the oxidation-reduction process (4 up and 7 down), and cation transport (1 down).

#### 61 Proteins were induced in response to both Cd and Zn stresses

We observed 61 DEPs whose relative abundances were altered in response to both Cd and Zn stresses (Figure 2, Data Sheet 8). However, under Cd stress, the relative abundances of 24 proteins were increased and 37 were decreased, whereas under Zn stress, 29 were increased and 32 were decreased. We also observed 12 proteins whose relative abundances were altered inversely in response to Cd and Zn stress (Data Sheet 8, marked by yellow). These proteins were not induced in response to combined Cd+Zn stress, indicating that they might be involved in the mutual inhibition of Cd/Zn. Among the 61 proteins we identified were 5 noteworthy proteins that we divided into three functional pathways (Table 1): The oxidation-reduction process (2), sucrose metabolism (1) and SAM metabolism (2).

#### 33 Proteins were induced in response to both Cd and Cd+Zn stresses

We identified 33 proteins whose relative abundances were induced in response to both Cd and combined Cd+Zn stresses (Figure 2, Data Sheet 9). Under Cd stress, 13 proteins were more abundant, and 20 were less abundant, whereas under Cd+Zn stress, 18 were more abundant, and 15 were less abundant. These results indicate that Cd and Cd+Zn stresses induce differential molecular responses. We also identified 11 proteins whose relative abundances were altered inversely in response to Cd and Cd+Zn, including nicotianamine synthase 2 (NAS; Data Sheet 9, marked by yellow). That these proteins were not induced in response to Zn stress suggests that they might participate in Cd detoxification of Zn. Among the 33 proteins we identified, 2 proteins were key enzymes in SAM metabolism, 1 was involved in Ca metabolism, and 1 participated in sucrose metabolism (Table 1).

#### 57 Proteins were induced in response to both Zn and Cd+Zn stresses

We identified 57 proteins whose relative abundances were induced in response to both Zn and combined Cd+Zn stresses (Figure 2, Data Sheet 10). Under Zn stress, 28 proteins were more abundant, and 29 were less abundant, whereas under Cd+Zn stress, 39 were more abundant, and only 18 were less abundant (Data Sheet 10). These results indicate that Zn and Cd+Zn stresses induce differential molecular responses. We also identified 28 proteins whose relative abundances were altered inversely in response to Zn and Cd+Zn, including glutaredoxin-C8 and glucan 1,3-beta-glucosidase, (Data Sheet 10, marked by yellow). These proteins were not induced in response to Cd stress, which suggests that they might participate in Zn detoxification of Cd. Among the 57 proteins we identified, 4 proteins functioned in GSH metabolism, 3 were peroxidases involved in the oxidation-reduction process, and 1 was involved in sucrose metabolism (Table 1).

#### 47 Proteins were induced only in response to Cd+Zn stress

We identified 47 proteins whose relative abundances were induced only in response to Cd+Zn stress (Figure 2, Data Sheet 11). These proteins were not induced in response to either Cd or Zn stress alone, which suggests that the molecular response induced by Cd+Zn stress differs from that induced by Cd and Zn stresses individually. Of the 47 proteins we identified, 24 proteins were more abundant, and 23 proteins were less abundant (Data Sheet 11). Among these, we identified 11 noteworthy proteins that functioned in Ca metabolism, the oxidation-reduction process, organic acid metabolism, and sucrose metabolism (Table 1).

## Discussion

Interactions between Cd and Zn have previously been shown to be antagonistic and/or synergistic in various plants (Hart et al., 2002, 2005; Sun et al., 2005; Tkalec et al., 2014). In the present study, Cd uptake was inhibited by Zn and Zn uptake was inhibited by Cd in DPW roots (Figure 1). Cd transport from root to shoot was inhibited by Zn (Figure 1A) but was promoted by Zn after 5 days after treatment (unpublished data). Meanwhile, Zn transport from root to shoot was not affected by Cd (Figure 1B). These results indicate that Cd and Zinc interact antagonistically in DPW seedlings, as previously reported in bread and durum wheat (Hart et al., 2002, 2005; Sun et al., 2005) and unlike the synergistic interactions that have been reported in other wheat under field conditions (Nan et al., 2002).

Proteomic changes in the roots implicated several proteins in the antagonistic interactions of Cd/Zn (Data Sheets 1–11). Two days after treatment, the relative abundances of 206 (Data Sheet 2), 303 (Data Sheet 3), and 190 (Data Sheet 4) proteins were induced in response to Cd, Zn, and Cd+Zn stresses, respectively (Figure 2). Among these, 53 proteins were induced in response to all three treatments, and 58, 131, and 47 proteins were induced in response to only Cd, Zn, or Cd+Zn stresses, respectively (Figure 2). We grouped these proteins into different interactions of Cd/Zn (Figure 2). Our results indicate that although Cd and Zn have similar physical and chemical properties (Chesworth, 1991) and pathways for uptake (Grant et al., 1998), they induce differential molecular responses (Lin and Arats, 2012), which result in the antagonistic interactions of Cd/Zn in DPW roots (Figure 1) and the high tolerance of DPW to Cd and Zn toxicity (Wang X. et al., in press). Some proteins identified in this study that are involved in noteworthy processes are discussed below.

To overcome oxidative toxicity caused by heavy metal stresses (Ranieri et al., 2005; Lin et al., 2007; Kieffer et al., 2008; Di Baccio et al., 2011; Zeng et al., 2011), plants utilize an effective antioxidant system that protects their cells against oxidative damage (Kieffer et al., 2008; Di Baccio et al., 2011) by inducing the expression of oxidation-reduction-related proteins (Lin et al., 2007; Kieffer et al., 2008, 2009; Di Baccio et al., 2011; Zeng et al., 2011; Schneider et al., 2013). In this study, 31 oxidation-reduction-related proteins were observed (Table 1). Of these, 8, 11, and 4 proteins were altered in response to Cd, Zn, and Cd+Zn stresses, respectively (Table 1). These results suggest that Cd, Zn, and Cd+Zn stresses cause differential oxidative threats which are detoxified through the induction of different oxidation-reduction-related proteins. Conversely, 8 Cd-induced proteins, 11 Zn-induced proteins, and 2 proteins induced by both Cd and Zn stresses were not induced in response to combined Cd+Zn stress, which suggests that the oxidative threats caused by Cd and Zn stresses are not the same as those caused by Cd+Zn stress. These results indicate that Cd and Zn detoxify each other in combined Cd+Zn stress, resulting in their uptakes being inhibited by each other. As described in previous studies (Kieffer et al., 2008, 2009; Schneider et al., 2013), Cd and Zn induced a greater abundance of some oxidative stress-related proteins but also induced a lower abundance of other oxidative stress-related proteins (Table 1). Among these, 3 proteins were induced by all 3 stresses (Cd, Zn, and Cd+Zn) (Table 1), including ascorbate peroxidase (protein 285), L-ascorbate peroxidase 2 (protein 573), and peroxisome type ascorbate peroxidase (protein 511), which are key peroxide detoxification enzymes (Raven et al., 2004). These results suggest that ascorbate mediates Cd- and Zn-induced oxidative stress in plants (Kieffer et al., 2008).

In response to Cd and Zn stresses, plants form heavy metal-glutathione (GSH) or metal-phytochelation (PC) compounds for metal detoxification (Seth et al., 2012; Jozefczak et al., 2015). GSH metabolism-related proteins, such as glutathione S-transferase (GST) and glutaredoxin (Grx), are differentially induced by Cd or Zn stress (Ahsan et al., 2009; Alvarez et al., 2009; Kieffer et al., 2009; Smiri et al., 2011; Zeng et al., 2011; Schneider et al., 2013). Meanwhile, GSTs translocate compounds of GSH-cytotoxic substrates into vacuoles for detoxification (Kumar et al., 2013). In this study, all three stresses (Cd, Zn, and Cd+Zn) induced GST (protein 405), Grx (protein 141), lactoyglutathione lyase (proteins 424), and 2 sulfate metabolism-related proteins [sulfurtransferase (protein 540) and ATP sulfurylase (protein 112)] (Table 1), suggesting that sulfate availability for the synthesis of metal chelations such as GSH (Speiser et al., 1992) determines Cd and Zn tolerance (Nocito et al., 2006; Alvarez et al., 2009). Additionally, 3 GSH metabolism-related proteins, including glutamine synthetase cytosolic isozyme 1-2 (protein 210), GST (protein 696) and lactoyglutathione lyase (proteins 135), were induced only in response to Cd stress (Table 1), suggesting that Cd is detoxified through sequestration of GSH-Cd compounds into vacuoles and subsequent reduction of oxidative stress (Seth et al., 2012; Jozefczak et al., 2015). However, these proteins were not induced in response to combined Cd+Zn stress, which partly illustrates Zn detoxification of Cd. Interestingly, 2 GSTs (proteins 79 and 731) and 2 Grxs (proteins 384 and 472) were induced in response to both Zn and Cd+Zn stresses (Table 1), similar to the results obtained for some GSTs induced by Zn stress in *Noccaea caerulescens* (Schneider et al., 2013). These results suggest that these proteins participate in the detoxification of Zn stress-induced reactive oxygen species (Dixon et al., 2009; Schneider et al., 2013).

As a precursor of GSH, SAM plays important roles in protecting against Cd stress-induced reactive oxygen species (ROS) (Noriega et al., 2007). In the present study, protein levels of serine hydroxymethyltransferase (SHMT) and spermidine synthase 1, key enzymes in SAM metabolism, were altered in response to both Cd and Zn stresses, suggesting that SAM plays important roles in protecting against these stresses. S-adenosylmethionine synthase (SAMS) synthesizes SAM, which is a precursor of nicotianamine (NA) (Schneider et al., 2013). The protein level of nicotianamine synthase 2 (NAS), which synthesizes nicotianamine (NA) from SAM, increased in response to Cd stress. NA is an essential compound for cell-to-cell transport of Zn, Fe, and Cu (Takahashi et al., 2003; Klatte et al., 2009). However, the abundances of both SAMS and NAS decreased in response to combined Cd+Zn stress, whereas a previous report in *N. caerulescens* showed increased SAMS and NAS levels in response to Zn stress (Schneider et al., 2013). Our results partially illustrate Cd inhibition of Zn uptake. Cd stress also causes the lignification of roots (Finger-Teixeira et al., 2010). SAM provides the methyl donor to caffeic acid 3-O-methyltransferase (COMT) in lignin biosynthesis (Wang Y. et al., 2016). COMT levels were increased only in response to Cd stress (Table 1), suggesting that Cd also causes root lignification.

Some organic acids such as oxalate, malate, citrate, and fumarate are induced by Cd and Zn stress (Ueno et al., 2005; López-Millán et al., 2009; Zhu et al., 2011; Schneider et al., 2013) and form metal-organic acid complexes to act as metal chelators to promote detoxification in *planta* (Verbruggen et al., 2009). Further, Cd and Zn also induce key enzymes that participate in organic acid metabolism (López-Millán et al., 2009; Schneider et al., 2013). In this study, Zn stress induced several of these enzymes (Table 1), including malate dehydrogenase (protein 58), isocitrate dehydrogenase (protein 225), aconitate hygratase (protein 39), citrate synthase 4 (protein 82), and oxalate oxidase GF-2.8 (protein 376). These results suggest that detoxification of Zn could be achieved through the formation of Zn-organic acid complexes and subsequently, the complexes are deposited into vacuoles (Schneider et al., 2013). Conversely, organic acid secretion is associated with Cd and Zn exclusion (Zhu et al., 2011). Combined Cd+Zn stress resulted in decreased abundances of furmarate hydratase 2 (protein 116), malate dehydrogenase 1 (protein 537) and succinate dehydrogenase (815), which are key enzymes in furmarate, malate, and citrate metabolisms, respectively (Table 1). Thus, our results partially illustrate the mutually inhibited uptake of Cd/Zn in the roots (Figure 1).

Cellulose and pectic polysaccharides are major components of the plant cell wall (Cosgrove, 2005), which can be modified by some heavy metals. For example, Cd enhances the contents of glucose and polysaccharides in cell walls (Li et al., 2015). Further, exogenous glucose alleviates Cd toxicity by increasing Cd fixation in root cell walls (Shi et al., 2015). In this study, several sucrose metabolism-related proteins were induced by Cd, Zn, or Cd+Zn stress (Table 1), suggesting that glucose and/or polysaccharides participate in Cd and Zn fixation, exclusion or sequestration in root cell walls (Li et al., 2015; Shi et al., 2015). However, 8 sucrose metabolism-related proteins were induced in response to Zn stress but not combined Cd+Zn stress (Table 1), which suggests that Cd detoxifies excess Zn by inhibiting its uptake, resulting in Cd-induced inhibition of Zn modification of sucrose metabolism.

Zn stress also affects the expression of P-type ATPases and several other metal transporters (Schneider et al., 2013). P-type ATPases, such as AtHMA4 from *Arabidopsis*, GmHMA3 from soybean and AhHMA3 from *A. halleri*, have Zn uptake activity (Becher et al., 2004; Hussain et al., 2004; Wang et al., 2012). Further, AtHMA2, 3, and 4 have been shown to transport Zn from root to shoot (Williams and Mills, 2005). In this study, the abundance of a P-type proton pump ATPase (protein 679) decreased in response to Zn stress but not combined Cd+Zn stress (Table 1), a result which contradicts previous work in *N. caerulescens* showing increased abundance of two P-type ATPases in response to Zn stress (Schneider et al., 2013). However, we found that other Zn-induced metal transporters were not observed 2 days after treatment, but their transcripts were regulated by Cd, Zn, and Cd+Zn 5 days after treatment (unpublished data).

Finally, as reported by previous studies (Di Baccio et al., 2011; Zeng et al., 2011), proteins that were similarly or differentially induced in response to Cd, Zn, and/or Cd+Zn stresses also participated in other processes, including ribosome biogenesis, DNA metabolism, protein folding/modification (all SFiles), suggesting that these proteins might contribute to differential defense mechanisms against these stresses (Zeng et al., 2011).

## Conclusion

Taken together, our results indicate that Cd and Zn interact antagonistically in DPW seedlings. Although 206, 303, and 190 proteins were induced in response to Cd, Zn, and Cd+Zn stresses, respectively, only 53 proteins were induced in response to all three stresses. 58, 131, and 47 proteins were induced only in response to Cd, Zn, and Cd+Zn stresses, respectively (Figure 2). These proteins could be divided into groups that resulted in different Cd/Zn interactions. Our results suggest that Zn and Cd stresses cause differential molecular responses in DPW. Under these stresses, oxidative stress-related proteins, metal chelators, metabolism-related proteins, sucrose metabolism-related proteins, and metal transporters are differentially induced to participate in metal detoxification, which ultimately causes antagonistic interactions and enhanced tolerance of Cd and Zn.

## Author contributions

YW, XW, XX, and YZ conceived and designed research, and wrote the manuscript. YW, XW, XX, CW, FP, and RW conducted experiments. YW, XW, JZ, HK, XF, LS, and HZ analyzed data. All authors read and approved the manuscript.

### Conflict of interest statement

The authors declare that the research was conducted in the absence of any commercial or financial relationships that could be construed as a potential conflict of interest.

## Supplementary material

Data Sheet 1 **Information about proteins identified in this study**.

Data Sheet 2 **Proteins induced by Cd stress**.

Data Sheet 3 **Proteins induced by Zn stress**.

Data Sheet 4 **Proteins induced by Cd+Zn stress**.

Data Sheet 5 **Proteins induced by all three treatments**.

Data Sheet 6 **Proteins induced only by Cd stress**.

Data Sheet 7 **Proteins induced only by Zn stress**.

Data Sheet 8 **Proteins induced by both Cd and Zn stresses**.

Data Sheet 9 **Proteins induced by both Cd and Cd+Zn stresses**.

Data Sheet 10 **Proteins induced by both Zn and Cd+Zn stresses**.

Data Sheet 11 **Proteins induced only by Cd+Zn stress**.

## References

[B1] Ahsan N. Renaut J. Komatsu S. (2009). Recent developments in the application of proteomics to the analysis of plant responses to heavy metals. Proteomics 9, 2602–2621. 10.1002/pmic.200800935 19405030

[B2] Alvarez S. Beria B. M. Sheffield J. Cahoon R. E. Jez J. M. Hicks L. M. (2009). Comprehensive analysis of the *Brassica juncea* root proteome in response to cadmium exposure by complementrary proteomic approaches. Proteomics 9, 2419–2431. 10.1002/pmic.200800478 19343712

[B3] Balen B. Tkalec M. Šikić S. Tolić S. Cvjetko P. Pavlica M.. (2011). Biochemical responses of *Lemna minor* experimentally exposed to cadmium and zinc. Ecotoxicology 20, 915–826. 10.1007/s10646-011-0633-1 21416111

[B4] Becher M. Talke I. N. Krall L. Kramer U. (2004). Cross-species microarray transcript profiling reveals high constitutive expression of metal homeostasis genes in shoots of the zinc hyperaccumulator *Arabidopsis halleri*. Plant J. 37, 251–268. 10.1046/j.1365-313X.2003.01959.x 14690509

[B5] Broadley M. R. White P. J. Hammond J. P. Zelko L. Lux A. (2007). Zinc in plants. New phytol. 173, 677–702. 10.1111/j.1469-8137.2007.01996.x 17286818

[B6] Chesworth W. (1991). Geochemistry of micronutrients, in Micronutrients in Agriculture, 2nd Edn, eds Mortvedt J. J. Cox F. R. Shuman L. M. Welch R. M. (Madison, WI: Soil Science Society of America), 1–30.

[B7] Cosgrove D. J. (2005). Growth of the plant cell wall. Nat. Rev. Mol. Cell Biol. 6, 850–861. 10.1038/nrm1746 16261190

[B8] Di Baccio D. Galla G. Bracci T. Andreucci A. Barcaccia G. Tognetti R. (2011). Transcriptome analysis of *Populus* × *euramericana* clone I-214 leaves exposed to excess zinc. Tree Physiol. 31, 1293–1308. 10.1093/treephys/tpr106 22038866

[B9] Dixon D. P. Hawkins T. Hussey P. J. Edwards R. (2009). Enzyme activities and subcellular localization of members of the *Arabidopsis* glutathione transferase superfamily. J. Exp. Bot. 60, 1207–1218. 10.1093/jxb/ern365 19174456 PMC2657551

[B10] Finger-Teixeira A. Ferrarese Mde L. Soares A. R. da Silva D. Ferrarese-Filho O. (2010). Cadmium-induced lignification restricts soybean root growth. Ecotoxicol. Environ. Saf. 73, 1959–1964. 10.1016/j.ecoenv.2010.08.021 20817298

[B11] Fukao Y. Ferjani A. Tomioka R. Nagasaki N. Kurata R. Nishimori Y.. (2011). iTRAQ analysis reveals mechanisms of growth defects due to excess zinc in Arabidopsis. Plant Physiol. 155, 1893–1907. 10.1104/pp.110.169730 21325567 PMC3091079

[B12] Grant C. A. Buckley W. T. Bailey L. D. Selles F. (1998). Cadmium accumulation in crops. Can. J. Plant Sci. 78, 1–17. 10.4141/P96-10017619806

[B13] Hart J. Welch R. M. Norvell W. A. Clarke J. M. Kochian L. V. (2005). Zinc effects on cadmium accumulation and partitioning in near-isogenic lines of durum wheat that differ in grain cadmium concentration. New Phytol. 167, 391–401. 10.1034/j.1399-3054.2002.1160109.x 15998393

[B14] Hart J. Welch R. M. Norvell W. A. Kochian L. V. (2002). Transport interactions between cadmium and zinc in roots of bread and durum wheat seedlings. Physiol. Plant. 116, 73–78. 10.1111/j.1469-8137.2005.01416.x 12207664

[B15] Hassan M. J. Zhang G. Wu F. Wie K. Chen Z. (2005). Zinc alleviates growth inhibition and oxidative stress caused by cadmium in rice. J. Plant Nutr. Soil Sci. 168, 255–261. 10.1002/jpln.200420403

[B16] Herbette S. Taconnat L. Hugouvieux V. Piette L. Magniette M. Cuine S.. (2006). Genome-wide transcriptome profiling of the early cadmium response of *Arabidopsis* roots and shoots. Biochimie 88, 1751–1765. 10.1016/j.biochi.2006.04.018 16797112

[B17] Hussain D. Haydon M. J. Wang Y. Wong E. Sherson S. M. Young J. (2004). P-type ATPase heavy metal transporters with roles in essential zinc homeostasis in *Arabidopsis*. Plant Cell 18, 1327–1139. 10.1105/tpc.020487 15100400 PMC423219

[B18] Jozefczak M. Bohler S. Schat H. Horemans N. Guisez Y. Remans T.. (2015). Both the concentration and redox state of glutathione and ascorbate influence the sensitivity of arabidopsis to cadmium. Ann. Bot. 116, 601–612. 10.1093/aob/mcv075 26070641 PMC4577996

[B19] Karp N. A. Huber W. Sadowski P. G. Charles P. D. Hester S. V. Lilley K. S. (2010). Addressing accuracy and precision issues in iTRAQ quantization. Mol. Cell. Proteomics 9, 1885–1897. 10.1074/mcp.M900628-MCP200 20382981 PMC2938101

[B20] Kazantzis G. (2004). Cadmium, osteoporosis and calcium metabolism. Biometals 17, 493–498. 10.1023/B:BIOM.0000045727.76054.f3 15688852

[B21] Kieffer P. Dommes J. Hoffmann L. Hausman J. F. Renaut J. (2008). Quantitative changes in protein expression of cadmium-exposed poplar plants. Proteomics 8, 2514–2530. 10.1002/pmic.200701110 18563750

[B22] Kieffer P. Planchon S. Oufir M. Ziebel J. Dommes J. Hoffmann L.. (2009). Combining proteomics and metabolite analyses to unravel cadmium stress-response in poplar leaves. J. Proteome Res. 8, 400–417. 10.1021/pr800561r 19072159

[B23] Klatte M. Schuler M. Wirtz M. Fink-Straube C. Hell R. Bauer P. (2009). The analysis of Arabidopsis nicotianamine synthase mutants reveals functions for nicotianamine in seed iron loading and iron deficiency responses. Plant Physiol. 150, 257–271. 10.1104/pp.109.136374 19304929 PMC2675739

[B24] Kumar S. Asif M. H. Chakrabarty D. Tripathi R. D. Dubey R. S. Trivedi P. K. (2013). Expression of a rice *Lambda* class of glutathione S-transferase, *OsGST2*, in *Arabidopsis* provides tolerance to heavy metal and other abiotic stresses. J. Hazard. Mater. 248–249, 228–237. 10.1016/j.jhazmat.2013.01.004 23380449

[B25] Lanquar V. Ramos M. S. Lelievre F. Barbier-Brygoo H. Krieger-Liszkay A. Kramer U. (2010). Export of vacuolar manganese by ArNRAMP 3 and AtNRAMP4 is required for optimal photosynthesis and growth under manganese deficiency. Plant Physiol. 152, 1986–1999. 10.1104/pp.109.150946 20181755 PMC2850043

[B26] Li T. Tap Q. Shohag M. J. I. Yang X. Sparks D. L. Liang T. (2015). Root cell wall polysaccharides are involved in cadmium hyperaccumulation in *Sedum alfredii*. Plant Soil 389, 387–399. 10.1007/s11104-014-2367-3

[B27] Lin R. Wang X. Luo Y. Du W. Guo H. Yin D. (2007). Effects of soil cadmium on growth, oxidative stress and antioxidant system in wheat seedlings (*Triticum aestivum* L.). Chemosphere 69, 89–98. 10.1016/j.chemosphere.2007.04.041 17568654

[B28] Lin Y. F. Arats M. G. M. (2012). The molecular mechanism of zinc and cadmium stress response in plants. Cell. Mol. Life Sci. 69, 3187–3206. 10.1007/s00018-012-1089-z 22903262 PMC11114967

[B29] López-Millán A. F. Sagardoy R. Solanas M. Abadía A. Abadía J. (2009). Cadmium toxicity in tomato (*Lycopersicon esculentum*) plants grown in hydroponics. Environ. Exp. Bot. 65, 376–385. 10.1016/j.envexpbot.2008.11.010

[B30] Michalcová V. Dušinskŷ R. Sabo M. Beyroutiová M. A. Hauptvogel P. Ivaničocá Z. (2014). Taxonomical classification and origin of Kamut® wheat. Plant Syst. Evol. 300, 1749–1757. 10.1007/s00606-014-1001-4

[B31] Nan Z. Li J. Zhang J. Cheng G. (2002). Cadmium and zinc interactions and their transfer in soil-crop system under actual field conditions. Sci. Tot. Environ. 285, 187–195. 10.1016/S0048-9697(01)00919-6 11874041

[B32] Nocito F. F. Lanchilli C. Crema B. Fourcroy P. Davidian J. C. Sacchi G. A. (2006). Heavy metal stress and sulfate uptake in maize roots. Plant Physiol. 141, 1138–1148. 10.1104/pp.105.076240 16698905 PMC1489904

[B33] Noriega G. O. Balestrasses K. B. Batlle A. Tomaro M. L. (2007). Cadmium induced oxidative stress in soybean plants also by the accumulation of delta-aminolevulinic acid. Biometals 20, 841–851. 10.1007/s10534-006-9077-0 17216352

[B34] Ranieri A. Castagna A. Scebba F. Careri M. Zagnoni I. Predieri G. (2005). Oxidative stress and phytochelatin characterization and in bread wheat exposed to cadmium excess. Plant Physiol. Biochem. 43, 45–54. 10.1016/j.plaphy.2004.12.004 15763665

[B35] Raven E. L. Lad L. Sharp K. H. Mewies M. Moody P. C. (2004). Defining substrate specificity and catalytic mechanism in ascorbate peroxidase. Biochem. Soc. Symp. 71, 27–38. 10.1042/bss0710027 15777010

[B36] Satarug S. Baker J. R. Urbenjapol S. Haswell-Wlkins M. Reilly P. E. B. Williams D. J.. (2003). A global perspective on cadmium pollution and toxicity in non-occupationally exposed population. Toxicol. Lett. 137, 65–83. 10.1016/S0378-4274(02)00381-8 12505433

[B37] Schneider T. Persson D. P. Husted S. Schellenberg M. Gehrig P. Lee Y.. (2013). A proteomics approach to investigate the process of Zn hyperaccumulation in *Noccaea caerulescens* (J & C. Presl) F.K. Meyer. Plant J. 73, 131–142. 10.1111/tpj.12022 22974502

[B38] Seth C. S. Remans T. Keunen E. Jozefczak M. Glelen H. Opdenakker K.. (2012). Phytoextraction of toxic metals: a central role for glutathione. Plant Cell Environ. 35, 334–346. 10.1111/j.1365-3040.2011.02338.x 21486307

[B39] Shi Y. Z. Zhu X. F. Wang J. X. Li G. X. Zheng S. J. (2015). Glucose alleviates cadmium toxicity by increasing cadmium fixation in roots cell wall and sequestration into vacuole in *Arabidopsis*. J. Integr. Plant Biol. 57, 830–837. 10.1111/jipb.12312 25404058

[B40] Smiri M. Chaoui A. Rouhier N. Gelhaye E. Jacquot J. P. Ferjani E. E. (2011). Cadmium affects the glutathione/glutatedoxin system in germinating *Pea* seeds. Biol. Trace Element Res. 142, 92–105. 10.1007/s12011-010-8749-3 20552295

[B41] Speiser D. M. Abrahamson S. L. Banuelos G. Ow D. W. (1992). *Brassica juncea* produces a phytochelatin-cadmium-sulfide complex. Plant Physiol. 99, 817–821. 10.1104/pp.99.3.817 16669006 PMC1080550

[B42] Straif K. Benbrahim-Tallaa L. Baan R. Grossw Y. Secretan B. El Ghissassi F. (2009). A review of human carcinogene-part C: metals, arsenic, dusts, and fibers. Lancet Oncol. 10, 453–454. 10.1016/S1470-2045(09)70134-2 19418618

[B43] Sun Q. Wang X. R. Ding S. M. Yuan X. F. (2005). Effects of interactions between cadmium and zinc on phytochelation and glutathione production in wheat (*Triticum aestivum* L.). Environ. Toxicol. 20, 195–201. 10.1002/tox.20095 15793816

[B44] Takahashi M. Terada Y. Nakai I. Nakanishi H. Yoshimura E. Mori S.. (2003). Role of nicotianamine in the intracellular delivery of metals and plant reproductive development. Plant Cell 15, 1263–1280. 10.1105/tpc.010256 12782722 PMC156365

[B45] Thomine S. Wang R. Ward J. M. Crawford N. W. Schroeder J. I. (2000). Cadmium and iron transport by members of a plant metal transporter family in *Arabidopsis* with homology to Nramp genes. Proc. Natl. Acad. Sci. U.S.A. 97, 4991–4996. 10.1073/pnas.97.9.4991 10781110 PMC18345

[B46] Tkalec M. Štefanić P. P. Cvjetko P. Šikić S. Pavlica M. Balen B. (2014). The effects of cadmium-zinc interactions on biochemical response in tobacco seedlings and adult plants. PLoS ONE 9:e87582 10.1371/journal.pone.0087582 24475312 PMC3903775

[B47] Ueno D. M. J. F. Iwashita T. Zhao F. J. McGrath S. P. (2005). Identification of the form of Cd in the leaves of a superior Cd-accumulating ecotype of *Thlaspi caerulescans* using ^113^Cd-NMR. Planta 221, 928–936. 10.1007/s00425-005-1491-y 15883836

[B48] Verbruggen N. Hermans C. Schat H. (2009). Molecular mechanisms of metal hyperaccumulation in plants. New Phytol. 181, 759–776. 10.1111/j.1469-8137.2008.02748.x 19192189

[B49] Wang X. Wang C. Sheng H. Wang Y. Zeng J. Kang H. (in press). Transcriptome-wide identification expression analysis of ABC transporters in dwarf polish wheat under metal stresses. Biol. Plant.

[B50] Wang Y. Wang C. Zhang H. Yue Z. Liu X. Ji W. (2013). Genetic analysis of wheat (*Triticum aestivum* L.) and related species with SSR markers. Genet. Resour. Crop Evol. 60, 1105–1117. 10.1007/s10722-012-9907-6

[B51] Wang Y. Xiao X. Wang X. Zeng J. Kang H. Fan X.. (2016). RNA-Seq and iTRAQ reveal the dwarfing mechanism of dwarf polish wheat (*Triticum polonicum* L.). Int. J. Biol. Sci. 12, 653–666. 10.7150/ijbs.14577 27194943 PMC4870709

[B52] Wang Y. Xiao X. Zhang T. Kang H. Zeng J. Fan X. (2014). Cadmium treatment alters the expression of five genes at the Cda1 loucs in two soybean cultivars [*Glycine Max* (L.) Merr]. Sci. World J. 2014, 979750 10.1155/2014/979750 PMC4060588 24987750

[B53] Wang Y. Yu K. F. Poysa V. Shi C. Zhou Y. H. (2012). A single point mutation in GmHMA3 affects cadmium (Cd) translocation and accumulation in soybean seeds. Mol. Plant 5, 1154–1156. 10.1093/mp/sss069 22778157

[B54] Williams L. E. Mills R. (2005). P_1B_-ATPases- an ancient family of transition metal pumps with diverse functions in plants. Trends Plant Sci. 10, 491–502. 10.1016/j.tplants.2005.08.008 16154798

[B55] Wiwart M. Suchowilska E. Kandler W. Sulyok M. Groenwald P. Krska R. (2013). Can polish wheat (Triticum polonicum L.) be an interesting gene source for breeding wheat cultivars with increased resistance to *Fusarium* head blight? Genet. Resour. Crop Evol. 60, 2359–2373. 10.1007/s10722-013-0004-2

[B56] Wu Q. Yang A. Zou W. Duan Z. Liu J. Chen J. (2013). Transcriptional engineering of *Escherchia coli K4* for fructosylated chondroitin production. Biotechnol. Prog. 29, 1140–1149. 10.1002/btpr.1777 23804518

[B57] Yadav S. K. (2010). Heavy metals toxicity in plants: an overview on the role of glutathione and phytochelatins in heavy metal stress tolerance of plants. South Afr. J. Bot. 76, 167–179. 10.1016/j.sajb.2009.10.007

[B58] Zeng X. W. Qiu R. L. Ying R. R. Tang Y. T. Tang L. Fang X. H. (2011). The differentially-expressed proteome in Zn/Cd hyperaccumulator *Arabis paniculata* Franch. in response to Zn and Cd. Chemosphere 82, 321–328. 10.1016/j.chemosphere.2010.10.030 21074242

[B59] Zhu X. F. Zheng C. Hu Y. T. Jiang T. Liu Y. Dong N. Y. (2011). Cadmium-induced oxalate secretion from root apex is associated with cadmium exclusion and resistance in *Lycopersicon esculentum*. Plant Cell Environ. 34, 1055–1064. 10.1111/j.1365-3040.2011.02304.x 21388421

